# Molecular Characterization of Omega-3 Fatty Acid Desaturases Reveals Functional Conservation and Their Pivotal Role in Salt and Temperature Stress Adaptation in *Arabidopsis thaliana*

**DOI:** 10.3390/ijms27093877

**Published:** 2026-04-27

**Authors:** Sajeel Hussain, Ye-Song Kim, Ashim Kumar Das, Da-Sol Lee, Adil Hussain, Byung-Wook Yun

**Affiliations:** 1 Department of Applied Biosciences, College of Agriculture and Life Sciences, Kyungpook National University, Daegu 41566, Republic of Korea; sajeel01@knu.ac.kr (S.H.); ttoksongi@naver.com (Y.-S.K.); ashim@knu.ac.kr (A.K.D.); giftanna@naver.com (D.-S.L.); 2 Department of Agriculture, Abdul Wali Khan University, Mardan 23200, Pakistan

**Keywords:** omega-3 fatty acid desaturase, endoplasmic reticulum, chloroplast, in silico analysis, salt stress and temperature

## Abstract

Omega-3 fatty acid desaturases (FAD3, FAD7, and FAD8) are key enzymes responsible for the production of α-linolenic acid (ALA), which is an essential polyunsaturated fatty acid regulating membrane stability and serves as a precursor for jasmonic acid. In this study, we performed a comprehensive genome-wide molecular characterization of omega-3 fatty acid desaturase genes across seven plant species. Phylogenetic analysis placed FAD3 and FAD7/FAD8 proteins in distinct clades, indicating functional divergence despite strong sequence conservation. Gene structure analysis revealed conserved exon–intron formation with a few unique features. Multiple sequence alignment and motif analysis revealed high sequence similarity with three histidine-rich boxes responsible for catalytic activity. *Cis*-regulatory elements revealed the abundance of light-responsive and ABA-responsive elements such as Box4 and ABRE, suggesting environmentally induced responses of omega-3 fatty acid desaturase genes. Moreover, q-RT PCR analysis revealed that the combined stresses of temperature and salt strongly influence the transcript levels of omega-3 fatty acid desaturase (*FAD3*, *FAD7*, and *FAD8*) in *Arabidopsis thaliana*; among them, the *FAD8* gene displayed significantly higher expression levels under salt stress conditions, especially at 22 °C temperature, indicating its possible leading role in stress adaptation. Furthermore, comparative promoter analysis revealed enrichment of stress-responsive motifs in the promoter regions of *AtFAD7* and *AtFAD8*, whereas *AtFAD3* contained more ABA-responsive elements. In addition, Pearson correlation analysis revealed a temperature-dependent relationship between promoter motifs and gene expression under salt stress at 31 °C in *Arabidopsis thaliana*. Overall, these findings suggest that omega-3 fatty acid desaturases are highly conserved yet transcriptionally dynamic under environmental conditions. This study provides a foundation for future genetic and functional studies of omega-3 fatty acid desaturase genes aimed at enhancing stress adaptation by targeting and regulating fatty acid metabolism.

## 1. Introduction

Omega (ω)3 polyunsaturated fatty acids (PUFAs) are considered beneficial for humans and most animals as they are essential for cell membranes and ion homeostasis, in addition to working as precursors to various regulatory molecules. Among the fatty acids, α-linolenic acid (ALA, 18:3 cisΔ9,12,15/n-3), docosahexaenoic acid (DHA, 22:6n−3), and eicosapentaenoic acid (EPA, 20:5n−3) are the most important [1,2]. ALA is synthesized by the addition of a double bond to linoleic acid (LA, 18:2 cisΔ9,12/n−6), which is performed by the endoplasmic reticulum-localized and chloroplast-localized omega-3 fatty acid desaturase (FAD3, FAD7, and FAD8) enzymes [3,4,5,6]. While studying *Arabidopsis thaliana* FAD3, FAD7, and FAD8 enzymes, McConn et al. [7] demonstrated that all three omega-3 fatty acid desaturase enzymes contribute to the synthesis of ALA, which acts as a precursor for the biosynthesis of jasmonic acid (JA).

Alpha-linolenic acid (C18:3) is essential for human health; however, it can not be synthesized in the human body. Therefore, ALA is obtained from food to overcome its deficiency in the body [8]. In humans, further desaturation is stopped after the conversion of C18:0 to C18:1 due to the absence of desaturases [9,10]. ALA is converted into docosahexaenoic acid (C22:6, DHA) and eicosapentaenoic acid (C20:5, EPA) which play several beneficial functions in neurodevelopment and skeletal health, and in easing depression [11,12]. Additionally, DHA and EPA also act as protective agents against several cardiovascular disorders, obesity, cancer, and others [13,14,15,16,17,18,19].

In plants, JA biosynthesis is initiated in the chloroplast, where various sequentially activated enzymes play their role in the initial step of JA biosynthesis, including lipoxygenase (LOX), allene oxide cyclase (AOC), and allene oxide synthase (AOS), leading to the formation of 12-oxo-phytodienoic acid (12-OPDA) [20,21]. 12-OPDA is translocated from the chloroplast to peroxisome through CTS channels by the JASSY protein, where it is reduced to 3-oxo-2(29[Z]-pentenyl)-cyclopentane-1-octanoic acid (OPC:8), which then undergoes three rounds of β-oxidation to produce the phytohormone JA [22]. The JASSY protein is located in the chloroplast outer envelope membrane, which functions in the transportation of OPDA, while COMATOSE (CTS), is localized on the peroxisomal membrane, which imports the OPDA into the peroxisome. Once JA is synthesized, it is conjugated to the amino acid isoleucine (ILE) by the enzyme JAR1 to form JA-ILE, which is the bioactive form of the JA hormone [23]. JA-ILE is perceived by the receptor *COI1*, leading to the degradation of JAZ and activation of downstream genes [24]. JA-ILE is transported throughout the whole plant and is subsequently deactivated via hydroxylation by the cytochrome P450 enzyme [25].

As described by [26], the *fad7* knockout mutant of *Arabidopsis thaliana* results in a 29% reduction in 18:3 content. In contrast, the disruption of the *fad8* knockout mutant of *Arabidopsis thaliana* results in trienoic fatty acid levels almost similar to Col-0 when grown at 22 °C. Similar results were reported by McConn and Browse [27] when they compared the ALA (18:3) content synthesized by the *Arabidopsis thaliana* knockout mutants *fad8*, *fad7*, *fad3*, *fad3 × fad7*, *fad7 × fad8*, and triple mutant (*fad3 × fad8 × fad7*). The *fad7* knockout mutant had the lowest ALA content followed by *fad3,* and then the *fad8* knockout mutant, indicating a significant role of the *FAD7* enzyme in synthesizing ALA. Furthermore, reduced levels of ALA were observed in the double mutant of *fad7 × fad8* compared to *fad3 × fad7*. These findings suggest a predominant role of *fad7* in ALA production, whereas *fad3* and *fad8* produce nearly similar levels of ALA, with *fad3* producing slightly higher quantities than *FAD8*. In addition, the expression levels of the FAD7 gene are higher in the leaf tissues of *Arabidopsis thaliana* compared to the roots, based on GUS staining and qRT-PCR analysis, while *FAD3* is reported to be the major contributor to the synthesis of trienoic fatty acids in roots [28]. Moreover, Claessens [29] performed seed germination tests, fatty acid analyses, qRT-PCR, and membrane fluidity analyses on *Arabidopsis Col-0*, *fad3*, *fad7*, and *fad8*, and observed no phenotypic changes in terms of plant size, seed, or seed coat among the mutants and wild type Col-0; however, the *fad7* knockout mutant showed a slowed growth rate, and reduced levels of ALA content were noticed in *fad3*.

Temperature-dependent changes in the unsaturation of plant membrane lipids are controlled by ER-localized and chloroplast-localized ω-3 fatty acid desaturases. Plastidial ω-3 fatty acid desaturases *FAD8* transcript levels increase under low-temperature conditions in *Arabidopsis thaliana* shoots, and *FAD8* partially compensates for the disruption of *FAD7* at 22 °C temperature [26]. Moreover, ER-localized *FAD3* displayed enhanced accumulation in the root tips of wheat under low temperature (10 °C), which contributes to the higher production of α-linolenic acid content [30].

*Arabidopsis thaliana fad3-2 × fad7-3 × fad8* triple knockout mutant and Col-0 grown at 22 °C under 140 µmol m^−2^ s^−l^ continuous light showed no difference in the growth rate and phenotype [31]. Furthermore, evaluation of the Photosynthetic Fluorescence Parameters (F_v_/F_m_) also showed insignificant differences between Col-0 and *fad3-2 × fad7-3 × fad8* triple mutant. Similar results were reported by [32]. Additionally, similar results were reported in the case of *Glycine max* by [33] when the plant height of mock-inoculated, *FAD3*-silenced, vector-infected, and *FAD3* overexpression plants was compared under normal growth conditions. No significant differences were noticed in the chlorophyll content of *FAD3*-silenced, vector-inoculated, and mock-inoculated soybean plants. However, the roots/shoot biomass and the chlorophyll content of *FAD3*-overexpressing soybean plants were higher than those of *FAD3*-silenced, mock-inoculated, and vector-infected soybean plants under control, salinity, and drought stress conditions.

Possible differences in the development of pollen of wild type, *fad3*, *fad7*, *fad8* single mutants, and *fad3-2 × fad7-3 × fad8* triple mutant were examined, which revealed normal pollen development up to the tricellular stage; pollen in *fad3*, *fad7,* and *fad8* were non-viable with only 0.6% germination frequency. Moreover, *fad3-2 × fad7-3 × fad8* triple mutant pollen tubes were less than one-third of the length of WT pollen tubes, indicating a highly reduced fertility rate. Exogenous application of ALA and JA was performed to restore the pollen viability, reduced fertility, and seed set of the triple mutant [31]. The above literature indicates that the loss of function of omega-3 fatty acid desaturase genes (*FAD3*, *FAD7*, and *FAD8*) does not markedly have a significant impact on the overall plant phenotype; these genes play a crucial role in the reproduction, fertility, and phytohormone production in plants.

This study is a holistic genome-wide in silico analysis of the omega-3 fatty acid desaturase genes in different plant species to investigate the genome-wide similarities and differences of ER-localized and chloroplast-localized omega-3 fatty acid desaturase genes, which are responsible for the synthesis of alpha-linolenic acid (18:3), which acts as a precursor for jasmonic acid biosynthesis. Furthermore, we also report our novel findings via quantitative real-time PCR analysis to determine the salt and temperature combined stress transcriptional regulation of omega-3 fatty acid desaturase genes in *Arabidopsis thaliana*.

## 2. Results

### 2.1. Phylogeny and Gene Structure Analyses of FAD3, FAD7, and FAD8 Genes from Different Plant Species

**Figure 1:**
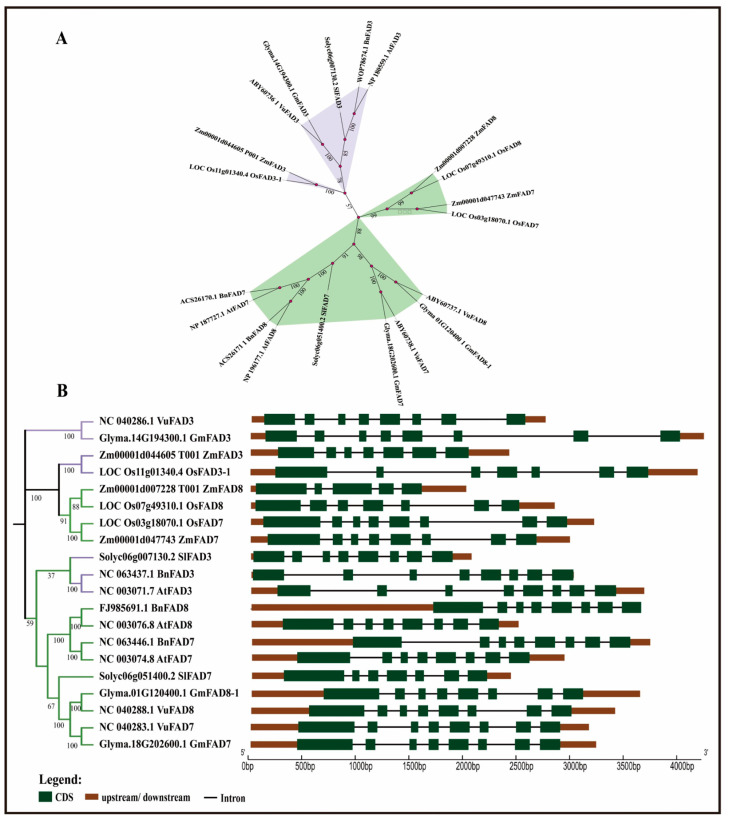
Phylogeny and gene structure analyses of *FAD3*, *FAD7*, and *FAD8* genes in different plant species. (**A**) The evolutionary relationship of omega-3 fatty acid desaturase genes was determined using the Neighbor-Joining method [34] with 1000 Bootstraps. The FAD3 proteins (ER-localized) of the selected plant species are highlighted in purple, while FAD7 and FAD8 (chloroplast-localized) of the selected plant species are highlighted in green. (**B**) Gene structure analysis revealed the number, positions, and sizes of introns and exons in the *FAD3*, *FAD7*, and *FAD8* genes of the selected plant species. Green boxes indicate exons, the black line indicates introns, and the promoters are shown in brown. The basepair scale is used to determine the sizes of exons and introns. The traditional phylogenetic tree shows the evolutionary relationship of ER-localized and chloroplast-localized omega-3 fatty acid desaturases. Branch color: green indicates chloroplast-localized, and purple indicates ER-localized omega-3 fatty acid desaturase genes.

The phylogenetic analysis was carried out using FAD3, FAD7, and FAD8 protein sequences from seven different plant species, downloaded from NCBI (https://www.ncbi.nlm.nih.gov/, accessed on 7 December 2025). Analysis indicated that all three omega-3 fatty acid desaturases are closely related with high bootstrap values, as shown in Figure 1A. Endoplasmic reticulum-localized omega-3 fatty acid desaturases are highlighted in purple, while chloroplast-localized omega-3 fatty acid desaturases are highlighted in green. The FAD3 protein of the selected plant species was clustered in a separate clade, whereas FAD7 and FAD8 were grouped together in a separate clade, indicating the different cellular localizations of ER-localized FAD3 and chloroplast-localized FAD7 and FAD8. The gene structures of the *FAD3*, *FAD7*, and *FAD8* genes were constructed on the Gene Display Server. Analysis indicated that omega-3 fatty acid desaturase genes (*FAD3*, *FAD7*, and *FAD8*) in most of the plant species has eight exons and seven introns, except *SlFAD7* (Solyc06g051400.2), *OsFAD8* (LOC_Os07g49310.1), *OsFAD3-1* (LOC_Os11g01340.4), and *ZmFAD3* (Zm00001d044605), which have seven exons and six introns (Figure 1). On the other hand, *ZmFAD8* (Zm00001d007228) has five exons and four introns. Among these, *GmFAD3* (Glyma.14G134300.1), *OsFAD3-1* (LOC_Os11g01340.4), and *AtFAD3* (NC_003071.7) have introns with longer lengths compared to omega-3 fatty acid desaturase genes in other plant species, as shown in Figure 1B.

### 2.2. Multiple Sequence Alignment and the Identification of Conserved Regions

**Figure 2:**
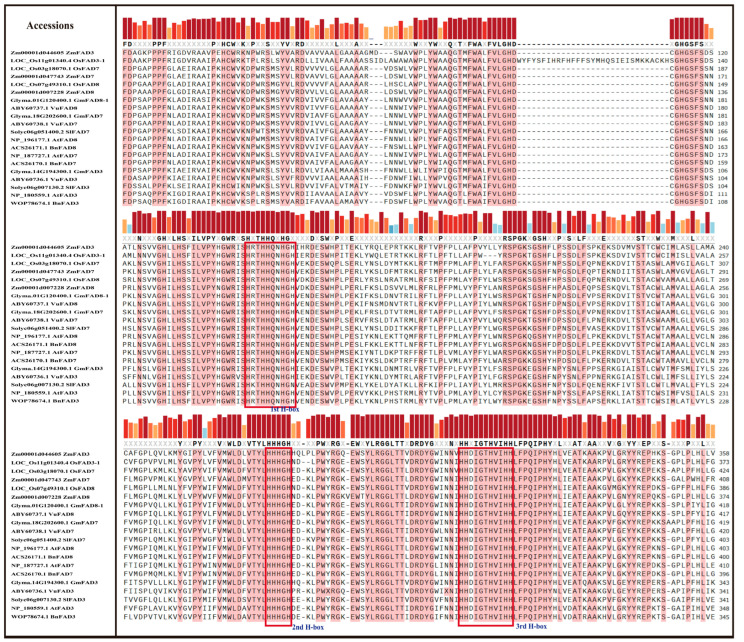
Multiple sequence alignment and the identification of conserved regions. Multiple alignment of FAD3, FAD7, and FAD8 in the selected plant species. Conserved regions in the amino acid sequence are highlighted in pink with red bars on top, whereas three highly conserved histidine boxes are enclosed in red boxes.

The amino acid sequences of the FAD3, FAD7, and FAD8 proteins of the selected plant species showed high sequence similarity, as shown in Figure 2, with conserved regions highlighted in pink. Despite the fact that FAD3 is localized in the ER and FAD7 and FAD8 are localized in the plastid, their amino acid sequences are still highly similar in the selected plant species. The two largest conserved regions are nine and sixteen amino acids long, and three histidine-rich motifs were also identified in the multiple-sequence alignment (enclosed in a red rectangle), as shown in Figure 2. These three histidine-rich boxes contain four to five histidine residues, which provide a molecular basis for the amino acid sequence similarity among the FAD3, FAD7, and FAD8 proteins.

### 2.3. Identification of Conserved Motifs in the Amino Acid Sequences of FAD3, FAD7, and FAD8

**Figure 3:**
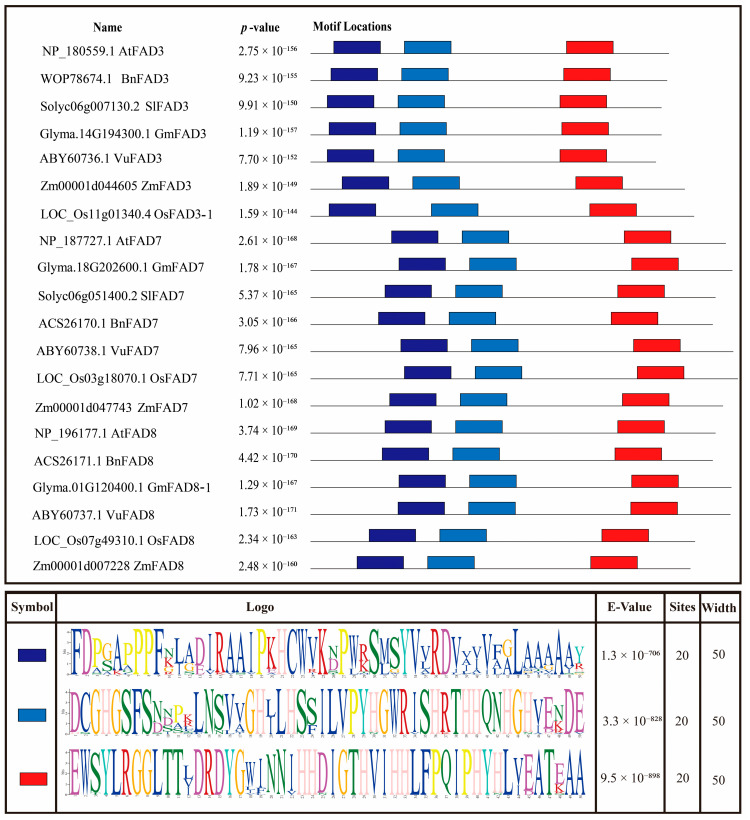
Identification of conserved motifs in the amino acid sequence of FAD3, FAD7, and FAD8 in the selected different plant species. Three highly conserved motifs were found in the protein sequence of FAD3, FAD7, and FAD8, shown in dark blue, blue, and red colors.

The motif analysis revealed three highly conserved motifs in the protein sequence of FAD3, FAD7, and FAD8 in the selected plant species (5′ dark blue, light blue, and orange 3′). The width and site of all three motifs were recorded as 50 and 20 with E-values, dark blue (1.3 × 10^−706^), light blue (3.3 × 10^−828^), and orange (9.5 × 10^−898^) as shown in Figure 3.

### 2.4. Predicted 3D Protein Structures

**Figure 4:**
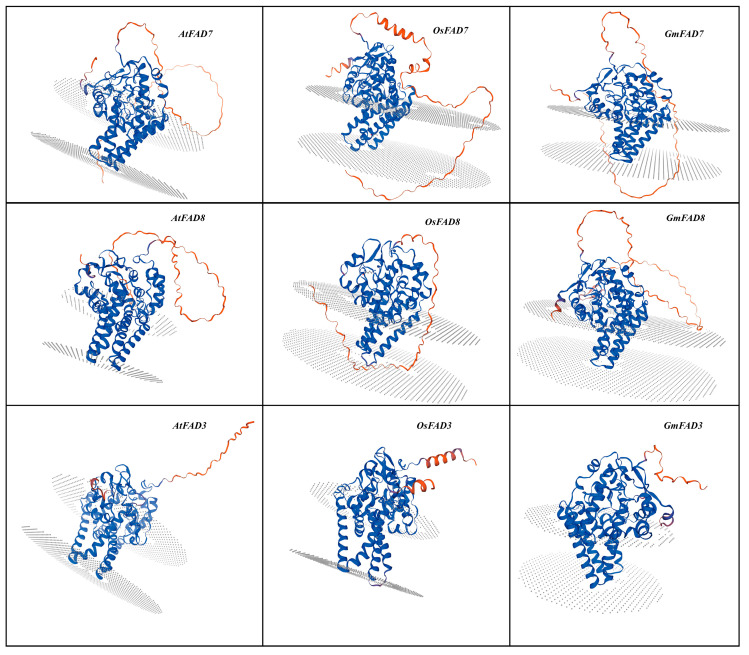
Predication of FAD3, FAD7, and FAD8 3D protein structures. The predicted 3D protein structure of FAD3, FAD7, and FAD8 of *Arabidopsis thaliana*, *Glycine max,* and *Oryza sativa*. Different colors indicate the confidence levels, with blue color indicating a range from 0.98 to 0.99, purple (0.69 to 0.74), and orange (0.39 to 0.43).

The predicted 3D protein structures of FAD3, FAD7, and FAD8 from *Arabidopsis thaliana*, *Glycine max*, and *Oryza sativa* exhibited highly significant structural similarity. All three omega-3 fatty acid desaturase proteins have an α-helical fold. In the case of FAD7 and FAD8, strong structural similarity was noticed in the central catalytic domains (blue) in *Arabidopsis thaliana*, *Glycine max,* and *Oryza sativa.* However, in all three omega-3 fatty acid desaturases, structural variation was observed in the loop and terminal regions (orange), as shown in Figure 4. In comparison, the FAD3 protein showed a slightly more compact structure than FAD7 and FAD8 across the three selected plant species.

### 2.5. Identification of Cis-Regulatory Elements

**Figure 5:**
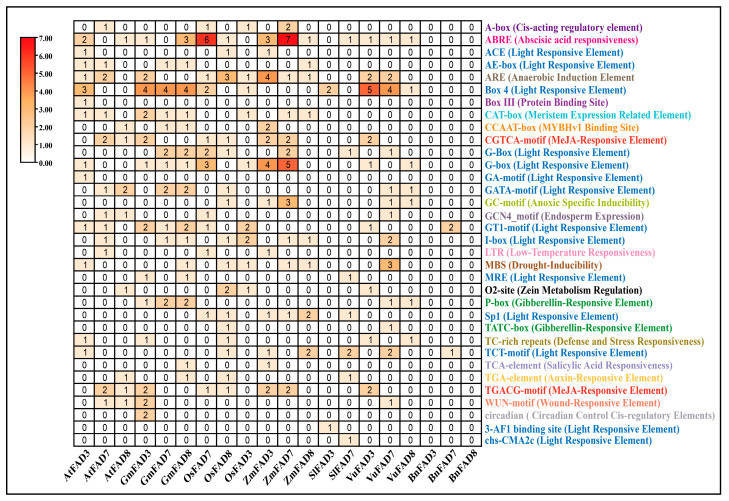
Identification of cis-regulatory elements in ER-Localized and chloroplast-localized omega-3 fatty acids desaturases promoters. Cis-regulatory elements were identified in a 1-kb promoter region of omega-3 fatty acid desaturase genes across the plant species studied. Cis-regulatory elements responsible for the same function are written in the same color. The heatmap frequencies range from 0 (white) to 7 (red).

The cis-regulatory element composition in the promoter regions of *FAD3*, *FAD7,* and *FAD8* genes in different plant species were identified. Various cis-regulatory elements were noticed in the promoter regions of ER-localized omega-3 fatty acid desaturases and chloroplast-localized omega-3 fatty acid desaturases across different plant species, among which light-responsive motifs were the most abundant category across all promoters. Elements such as Box 4, G-box, GT1-motif, I-box, GATA-motif, MRE, Sp1, and TCT-motif were widely distributed, indicating that these genes are strongly regulated by light signaling pathways. Among hormone-responsive elements, ABRE (ABA-responsive element) showed higher heatmap frequency, especially in *ZmFAD7* and *OsFAD7*. Moreover, MeJA-responsive elements such as the CGTCA-motif and TGACG-motif were also uniformly present in most of the promoter regions of omega-3 fatty acid desaturase, as shown in Figure 5.

### 2.6. Protein Interaction and Molecular Function Enrichment Analysis

**Figure 6:**
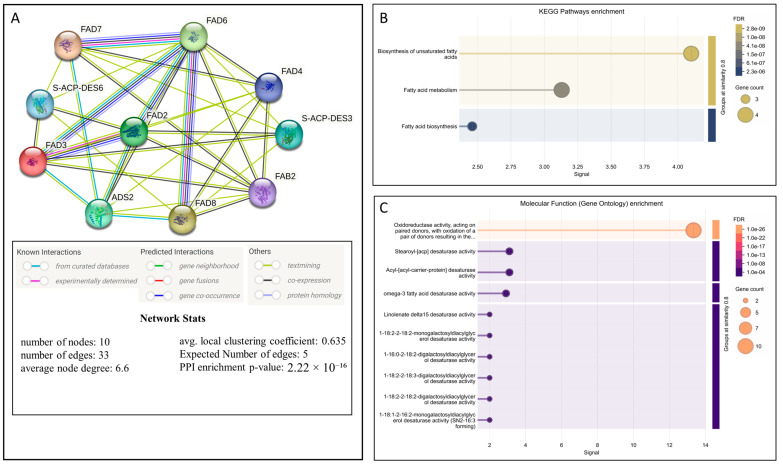
Protein interaction and molecular function enrichment analysis. (**A**) The interactions of omega-3 fatty acid desaturase proteins, including FAD6, FAD2, and neighboring proteins, in *Arabidopsis thaliana*, with a medium confidence level of 0.400. (**B**) The KEGG pathways analysis demonstrating the involvement of omega-3 fatty acid desaturases proteins in different pathways. (**C**) The predicted molecular function of omega-3 fatty acid desaturases and other genes with a similarity value of ≥0.8.

The protein–protein interaction (PPI) analysis showed a highly interconnected network among FAD3, FAD7, FAD8, FAD2, and FAD6 in *Arabidopsis thaliana*. The PPI network contained 22 edges, 10 nodes with an average node degree of 6.6 and an average local clustering coefficient of 0.635.0. FAD6 appears to be playing a major role in connecting pathways of plastidial and extraplastidial desaturation, suggesting an integrative regulatory role, as shown in Figure 6A. KEGG pathway enrichments reveal a higher representation of unsaturated fatty acid biosynthesis, followed by fatty acid metabolism and fatty acid biosynthesis, as shown in Figure 6B, confirming that these networks are tightly linked to lipid metabolism. The low FDR values and enrichment signals indicate that these associations are statistically accurate and biologically meaningful. Furthermore, gene ontology (Molecular Function) enrichment analysis reveals that the most significantly enriched term is oxidoreductase activity acting on paired donors, with oxidation of a pair of donors resulting in the reduction of molecular oxygen followed by stearoyl-(acp) desaturase activity, acyl (acyl-carrier-protein) desaturase activity, and omega-3 fatty acid desaturase activity, which confirms that the network is dominated by lipid desaturation enzymes as shown in Figure 6C.

### 2.7. Determination of Physico-Chemical Properties of FAD3, FAD7 and FAD8 Proteins

The physico-chemical properties of ER-localized (FAD3) and chloroplast-localized (FAD7 and FAD8) proteins were determined using the ProtParam database Bioinformatics Resource of the Portal (https://web.expasy.org/protparam/, accessed on 7 December 2025), which includes molecular weight, instability index, hydropathicity (GRAVY), aliphatic index, length, and pI as shown in Table 1. *VuFAD8* and *VuFAD3* had the highest and lowest molecular weights, 51,395.78 (da) and 43,234.05 (da), respectively. OsFAD7 had the highest instability index, length, and pI, with ratios of 53.55, 459, and 9.66, respectively. The lowest values were recorded in AtFAD3, VuFAD3, and VuFAD8, at 26.76, 371, and 7.13, respectively. Maximum aliphatic index and hydropathicity (GRAVY) were observed for AtFAD3 and AtFAD8, with values of 92.41 and −0.318, respectively. The minimum aliphatic index and hydropathicity (GRAVY) were observed for AtFAD8 and ZmFAD8.

### 2.8. Early Transcriptional Response of Arabidopsis FAD3, FAD7, and FAD8 to Combined Salt and Temperature Stress

**Figure 7:**
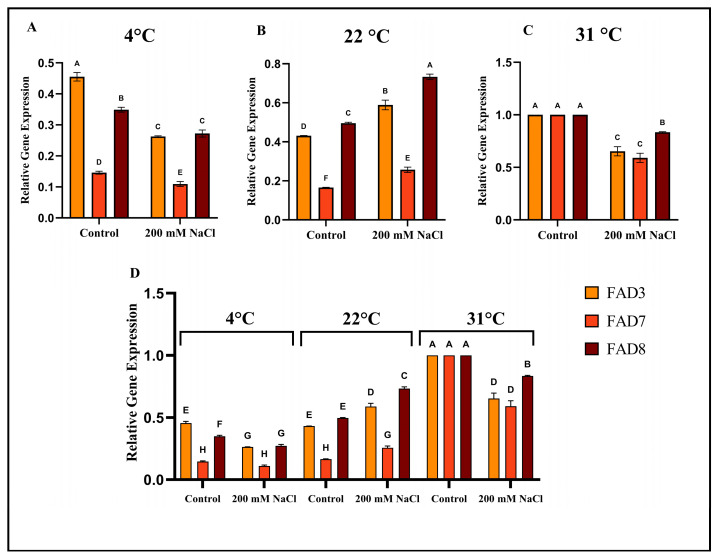
Transcriptional response of *Arabidopsis FAD3*, *FAD7*, and *FAD8* to combined salt and temperature stress. (**A**) Quantitative real-time PCR analysis of ER-localized and chloroplast-localized omega-3 fatty acid desaturase in *Arabidopsis thaliana* Col-0 was performed under combined 4 °C, (**B**) 22 °C, and (**C**) 31 °C conditions with 0 mM and 200 mM salt stress for 8 h. (**D**) shows the combined expression levels of *FAD3*, *FAD7*, and *FAD8* in all three temperatures under control and salt stress conditions. Each data point shows the mean of three independent replicates. Standard deviations are indicated by the error bars. Data were analyzed using two-way ANOVA followed by Tukey’s multiple comparison test at *p* < 0.05. Different letters on the bars indicate significant differences between the means.

The expression levels of ER-localized and chloroplast-localized omega-3 fatty acid desaturases in *Arabidopsis thaliana* Col-0 were evaluated at 4 °C, 22 °C, and 31 °C, and under 0 mM and 200 mM salt stress after 8 h of temperature and salt stress application (Figure 7). Quantitative real-time PCR analysis revealed that under control conditions (0 mM NaCl) at 4 °C, *FAD3* expression remained significantly higher, followed by *FAD8*, whereas *FAD7* showed significantly low transcript abundance, as shown in Figure 7A. However, in the absence of salt stress, an ambient temperature of 22 °C led to significant increases in the expression levels of all three genes, with *FAD8* exhibiting the highest expression, followed by *FAD3* and *FAD7,* as shown in Figure 7B. At 31 °C, all three genes exhibited similar expression levels under 0 mM salt stress, as shown in Figure 7C. In the case of 200 mM salt stress, the expression levels of all three genes were reduced compared to the control, with *FAD7* significantly down-regulated, whereas *FAD3* and *FAD8* showed moderate reductions in their expression levels at 4 °C. In contrast, under 200 mM salt stress and 22 °C temperature, the transcript levels of *FAD3*, *FAD7*, and *FAD8* were significantly induced compared to the control group, with *FAD8* displaying significantly higher expression levels, followed by *FAD3* and *FAD7*. However, at 31 °C, the expression levels of all three genes dropped, with *FAD3* and *FAD7* displaying reduced levels, though *FAD8* remained higher than *FAD3* and *FAD7* under salt stress conditions. In addition, the expression levels of omega-3 fatty acid desaturase genes in *Arabidopsis thaliana* remained significantly higher under control conditions at 31 °C temperature as compared to the control group of 4 °C and 22 °C temperatures, with *FAD8* maintaining significantly higher expression levels under 200 mM salt stress condition at both 22 °C and 31 °C temperatures as shown in Figure 7D. These results indicate a clear temperature-dependent regulation of omega-3 fatty acid desaturase gene expression resulting in the suppression of omega-3 fatty acid desaturases under salt stress conditions at 4 °C and 31 °C temperatures. In contrast, at ambient 22 °C temperature, salinity induced the omega-3 fatty acid desaturases transcription, with *FAD8* exhibiting the strongest response to the temperature and salt interaction level.

### 2.9. Identification of Conserved Motifs in the Promoter Region of AtFAD3, AtFAD7, and AtFAD8 and Their Comparative Analysis with q-RT PCR Data

**Figure 8:**
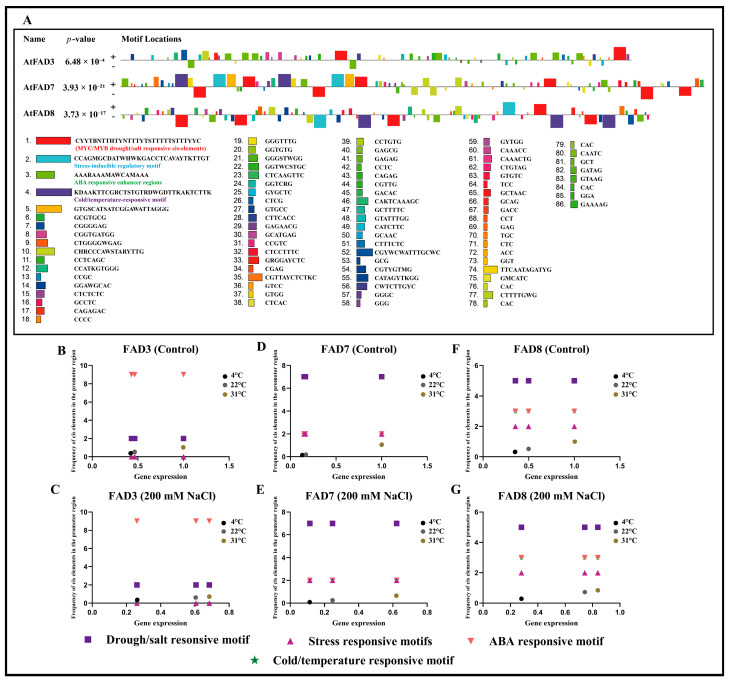
(**A**) The identification of conserved motifs in the promoter region of *AtFAD3*, *AtFAD7*, and *AtFAD8*. Among them, MYC/MYB drought/salt-responsive elements, stress-inducible regulatory motifs, ABA-responsive enhancer regions, and cold/temperature-responsive motifs are highlighted in red, blue, green, and purple, respectively. (**B**–**G**) The comparative relationship of identified promoter motifs with q-RT PCR data of *AtFAD3*, *AtFAD7*, and *AtFAD8* under control and 200 mM salt stress conditions. A scatter plot for comparative analysis was constructed using correlation analysis for XY data. Color dots show the gene expression data (black: 4 °C, gray: 22 °C, and brown: 31 °C).

The motif analysis of *Arabidopsis thaliana FAD3*, *FAD7,* and *FAD8* gene promoter regions revealed several conserved motifs such as MYC/MYB drought/salt-responsive elements, stress-inducible regulatory motifs, ABA-responsive enhancer regions, and cold/temperature-responsive motifs. Among these, drought/salt-responsive and stress-inducible motifs were more enriched in *AtFAD7* and *AtFAD8*, whereas *AtFAD3* contained a higher number of ABA-responsive elements. Notably, *AtFAD8* exhibited the greatest abundance of cold/temperature-responsive motifs compared to *AtFAD3* and *AtFAD7*, as shown in Figure 8A. To link the promoter motifs with the qRT-PCR data, a comparative analysis was conducted, which revealed that at 31 °C temperature under 0 mM salt stress conditions, *AtFAD3* expression was highest, in the presence of abundant ABA-responsive and drought/salt-related elements, while reduced expression was observed at 4 °C and 22 °C. Under 200 mM salt stress conditions, a higher expression of *AtFAD3* was observed both at 22 °C and 31 °C temperatures, while the expression levels remained low at 4 °C temperature in the presence of a high number of ABA-responsive enhancer regions and drought/salt-responsive motifs, as shown in Figure 8B,C. In the case of *AtFAD7*, low expression levels were observed at both 4 °C and 22 °C temperatures at 0 mM salt stress conditions, while a higher expression level was observed at 31 °C temperature in the presence of a high number of drought/salt-responsive motifs and a moderate number of ABA-responsive enhancer regions, stress-inducible regulatory motifs, and cold/temperature-responsive motifs. Moreover, the expression level of *AtFAD7* remained higher at 31 °C under 200 mM salt stress, followed by 22 °C and then 4 °C, as shown in Figure 8D,E. Similarly, under 0 mM salt stress conditions, *AtFAD8* had the highest expression levels at 31 °C, followed by 22 °C and then 4 °C in the presence of a high number of drought/salt-responsive motifs, ABA-responsive enhancer regions, and cold/temperature-responsive motifs. However, under 200 mM salt stress conditions, the expression level of *AtFAD8* remained higher at 31 °C, followed by an increase in the expression level of *AtFAD8* at 22 °C. Meanwhile, the expression of *AtFAD8* remained lower at 4 °C, as shown in Figure 8F,G. This suggests that the promoter motifs perform their best function at high temperature under both control and salt stress conditions.

### 2.10. Pearson Correlation Analysis of Promotor Motifs of AtFAD3, AtFAD7, and AtFAD8 and q-RT PCR Data

**Figure 9:**
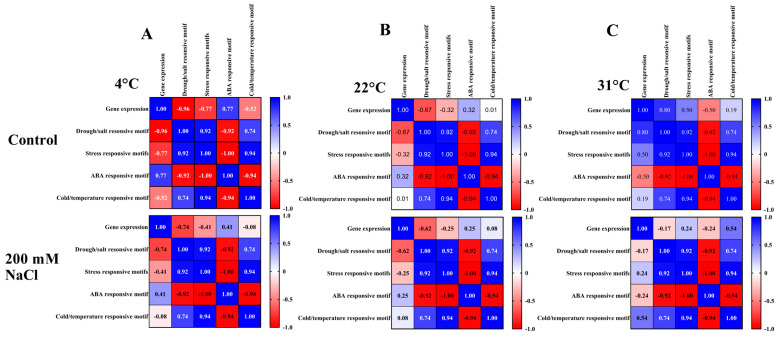
(**A**) Pearson correlation analysis of drought/salt-responsive, ABA-responsive, stress-related, and temperature-responsive motifs with q-RT PCR data under control and 200 mM NaCl at 4 °C. (**B**) Correlation analysis of drought/salt-responsive, ABA-responsive, stress-related, and temperature-responsive motifs with q-RT PCR data under control and 200 mM NaCl at 22 °C. (**C**) Correlation analysis of drought/salt-responsive, ABA-responsive, stress-related, and temperature-responsive motifs with q-RT PCR data under control and 200 mM NaCl at 31 °C. Bar shows the Pearson correlation r values ranging from −1.0 to 1.0 (red to blue).

The Pearson correlation analysis revealed various temperature- and stress-dependent relationships between the identified promoter motifs (Figure 8A) and the q-RT PCR data of *AtFAD3*, *AtFAD7*, and *AtFAD8* under control and 200 mM salt stress conditions. Under control conditions at 4 °C, the gene expression displayed a highly negative correlation with drought/salt-responsive motifs (−0.96) and stress-responsive motifs (−0.77), while exhibiting a positive correlation with ABA-responsive elements (0.77) at 4 °C temperature, as shown in Figure 9A. This provides support to the idea that cold conditions favor ABA-mediated regulation while suppressing drought- and stress-related motifs associated with regulation. At 22 °C, under control conditions, gene expression displayed moderate negative correlation with drought/salt-responsive motifs (−0.67) and weak associations with other motifs, indicating a reduced regulatory influence of these cis-elements under optimal growth temperature, as shown in Figure 9B. In contrast, under control conditions at 31 °C, an increase in the correlation values was observed, where gene expression displayed positive correlation with drought/salt-responsive motifs (0.80) and stress-responsive motifs (0.50), while showing a negative correlation with ABA-responsive elements (−0.50). This indicates the activation of stress-adaptive regulatory mechanisms under heat conditions. However, under 200 mM NaCl treatment, the correlations were generally reduced across all temperatures. At 4 °C and 22 °C, gene expression maintained moderate negative correlations with drought/salt-responsive motifs (Figure 9A,B), while at 31 °C, gene expression showed a positive correlation with cold/temperature-responsive motifs (0.54), suggesting a possible connection between salt and temperature stress pathways, as shown in Figure 9C.

## 3. Discussion

Alpha-linolenic acid (ALA), an omega-3 polyunsaturated fatty acid (PUFA), is considered to be an important component of cell membranes and acts as a vital precursor for jasmonic acid biosynthesis in plants [35]. It helps maintain membrane fluidity and integrity, which is essential for photosynthetic processes and overall plant vitality, mainly under stress conditions. ER-localized FAD3 and chloroplast-localized FAD7/FAD8 are leading enzymes that function in converting linoleic acid (LA) to ALA (18:3). These enzymes are located in different parts of the cell and often exhibit different regulatory patterns [36]. This study analyzed omega-3 fatty acid desaturase genes (*FAD3*, *FAD7*, and *FAD8*) in *Arabidopsis thaliana*, *Oryza sativa*, *Solanum lycopersicum*, *Zea mays*, *Brassica napus*, *Glycine max*, and *Vigna unguiculata* using high-throughput in silico techniques to characterize them. Phylogenetic analysis revealed a clear separation of the *FAD3* and *FAD7*/*FAD8* clades, with high bootstrap values, suggesting that they share strong sequence similarity and a common ancestor. This separation of *FAD3* and *FAD7*/*FAD8* clades reflects their subcellular localization and function, which agrees with the earlier functional and molecular characterization that ER-localized and chloroplast-localized ω-3 desaturases evolved distinct targeting sequences while maintaining conserved catalytic cores [37,38,39]. The close clustering of *FAD7* and *FAD8* can be explained by their shared evolutionary origin and high protein sequence homology, despite both genes encoding plastidial omega-3 fatty acid desaturases; *FAD7* and *FAD8* exhibit divergent responses at the regulatory level. *FAD7* and *FAD8* genes are likely to have arisen from a gene duplication event, which explains their close clustering in the conducted phylogenetic analysis. However, their naming reflects their specialized physiological contributions: *FAD7* is primarily responsible for the constitutive synthesis of trienoic fatty acids at ambient temperatures, while *FAD8* is specifically characterized by its temperature-sensitive regulation. Gene structure analysis displayed a high degree of conservation of ER-localized and chloroplast-localized omega-3 fatty acid desaturases, exhibiting mostly eight exons and seven introns, suggesting strong similarity across monocot and dicot lineages [38,40]. Multiple alignment also showed high sequence similarity among omega-3 fatty acid desaturase genes across the seven different plant species; and three histidine-rich regions were identified in all FAD3, FAD7, and FAD8 proteins. According to Xue et al. [38] and Yurchenko et al. [41], these histidine regions are essential for catalytic activity due to their binding with iron atoms, which are required for introducing double bonds in fatty acids. The identification of highly conserved motifs further supported the presence of conserved regions in the protein sequence. The predicted 3D protein structures displayed similar folding patterns, especially in the central catalytic regions; however, some variation was observed in the terminal and loop regions, which may contribute to differences in regulation or membrane interaction [42]. In addition, the conserved histidine-rich motifs common in all three omega-3 fatty acid desaturases are essential for catalytic activity and are located within the central region of the protein [37].

Moreover, cis-regulatory elements analysis revealed that light-responsive elements, ABA-responsive elements, and MeJA-responsive elements were the most abundant in the promoter regions of the omega-3 fatty acid desaturase genes in different plant species, which suggests that due to the presence of *FAD7*/*FAD8* in the chloroplast, they are closely linked to photosynthesis and are regulated by stress-related hormones [43,44,45]. Furthermore, ALA production can significantly affect JA production and, in turn, plant defense against a variety of stresses [46]. Therefore, transcriptional regulation of omega-3 fatty acid desaturase may directly influence stress signaling pathways. Protein–protein interaction analysis revealed that the FAD3, FAD7, FAD8, FAD6, and FAD2 proteins interact strongly with one another, with FAD6 serving as a key connector among these genes. These interactions indicate that omega-3 fatty acid desaturases play a significant role in the lipid metabolism network. Furthermore, KEGG enrichment analysis further confirmed that omega-3 fatty acid desaturase proteins are primarily involved in unsaturated fatty acid biosynthesis, supporting the concept that lipid metabolism and modification are interconnected and tightly controlled processes. The qRT-PCR analysis indicated that the expression of *FAD3*, *FAD7*, and *FAD8* genes in *Arabidopsis thaliana* Col-0 is strongly affected by temperature and salt conditions. At 22 °C, salt stress increases the expression of all three omega-3 fatty acid desaturases, especially *FAD8*. This suggests that at 22 °C, salt stress activates lipid desaturation to maintain membrane stability. At 4 °C and 31 °C, salt stress reduces the expression of all three omega-3 fatty acid desaturases, particularly *FAD7*. This suggests that the combined effects of extreme cold or heat, with salt stress, suppress the expression of omega-3 fatty acid desaturase genes. Previous studies reported that the *FAD8* gene expression is induced by low temperature to enhance membrane unsaturation [26], and *FAD3* protein production increases at low temperature in wheat roots [29]. Furthermore, the promoter motif analysis revealed that drought/salt-responsive and stress-inducible motifs are primarily enriched in *AtFAD7* and *AtFAD8*, while *AtFAD3* contains a higher number of ABA-responsive motifs. Additionally, *AtFAD8* revealed the highest abundance of cold/temperature-responsive motifs, which likely supports its unique role in temperature and salt stress adaptation compared to *AtFAD3* and *AtFAD7.* Comparative scatter plots revealed high expression levels at 31 °C, while lower expression was found at 4 °C and 22 °C under both control and salt stress conditions, suggesting the deactivation of these promoter motifs at low and normal temperatures, while activation occurs at the high temperature of 31 °C. Moreover, Pearson correlation analysis indicates that at 4 °C under normal conditions, a strong positive correlation was observed with ABA-responsive elements, suggesting the positive regulatory role of ABA-responsive elements under 4 °C temperature. Furthermore, under combined stress, a positive correlation was observed between cold/temperature-responsive motifs at 31 °C, which indicates a possible regulatory crosstalk, where temperature-responsive promoter motifs may contribute to regulating gene expression during salinity stress, suggesting an integrated stress-response mechanism. Our results suggest that when cold and hot temperatures are combined with salt stress, the regulatory mechanisms may differ from those under a single stress condition. However, under all three temperatures, under salt stress, the *FAD8* gene maintained higher expression levels than *FAD3* and *FAD7*, indicating that the *FAD8* gene tends to respond to early stress adaptation. Moreover, the expression levels of all three genes were higher under control conditions at 31 °C but reduced under salt stress conditions, which also suggests that when only 31 °C temperature is applied, the expression levels of all three omega-3 fatty acid desaturase genes are up-regulated as compared to the control group of 4 °C and 22 °C. Increased ALA production helps maintain fluidity and also supports JA biosynthesis, which is an essential hormone for stress responses.

## 4. Materials and Methods

### 4.1. Phylogenetic and Gene Structure Analysis

The phylogenetic tree was constructed using protein sequences of FAD3, FAD7, and FAD8 from 7 different plant species. Protein sequences were downloaded from Phytozome (https://phytozome-next.jgi.doe.gov/, accessed on 7 December 2025) and NCBI (https://www.ncbi.nlm.nih.gov/, accessed on 7 December 2025) on MEGA 11 software [34]. The tree was constructed using the Neighbor-Joining method, with 1000 Bootstraps, and was further modified in itol [47]. The *FAD3*, *FAD7*, and *FAD8* Genomic DNA and coding DNA sequences were downloaded from Phytozome (https://phytozome-next.jgi.doe.gov/) and NCBI (https://www.ncbi.nlm.nih.gov/) to determine gene structure on Gene Display Server [48].

### 4.2. Multiple Alignment and Identification of Conserved Regions

The FAD3, FAD7, and FAD8 protein sequences were aligned via Clustal W using EMBL-EBI (https://www.ebi.ac.uk/jdispatcher/msa, accessed on 19 February 2026). The downloaded multiple sequence alignment was viewed on SnapGene 8.1.0 at >95% sequence conservation level.

### 4.3. Motif Analysis

Downloaded protein sequences were used to find highly conserved motifs using MEME (Multiple EM for Motif Elicitation) (https://meme-suite.org/meme/index.html, accessed on 3 April 2026) [49]. Downloading all data SVG included information about motif width, site, and E-values.

### 4.4. Protein Structure Analysis

The predicted 3D structures of FAD3, FAD7, and FAD8 proteins in the selected plant species were constructed using SWISS-MODEL [50] by using the amino acid sequence downloaded from Phytozome (https://phytozome-next.jgi.doe.gov/) and NCBI (https://www.ncbi.nlm.nih.gov/).

### 4.5. Identification of Cis-Regulatory Elements

Cis-regulatory elements were identified in 1000 bp promoter sequences using the PlantCare Database (https://bioinformatics.psb.ugent.be/webtools/plantcare/html/, accessed on 20 February 2026) [51]. Data were used to construct a heatmap in TB Tools (v2.034) [52] displaying the frequencies of common cis-regulatory elements among *FAD3*, *FAD7*, and *FAD8* genes in the selected plant species.

### 4.6. Determination of Physicochemical Properties

Physicochemical attributes (including molecular weight, hydropathicity (GRAVY), instability index, length, and isoelectric point pI) of *FAD3*, *FAD7*, and *FAD8* proteins in different plant species were determined by using the ProtParam platform of the Swiss Bioinformatics Resource Portal ExPasy (https://web.expasy.org/protparam/, accessed on 7 December 2025) [53].

### 4.7. Determination of Protein–Protein Interaction, KEGG, and GO Enrichment Analysis

Protein–protein interactions, KEGG Pathway Enrichment, and Gene Ontology Enrichment Analysis of FAD3, FAD7, and FAD8 were determined by using STRING 12.0 (https://string-db.org/cgi/input?sessionId=bPIMptPaZthA&input_page_show_search=on, accessed on 25 February 2026).

### 4.8. Growth Conditions and RNA Extraction

Seeds of *Arabidopsis thaliana* Col-0 were surface sterilized and germinated in soil. After 14 days, seedlings were transplanted to pots of size 6 cm length and 6 diameter. Three plants per pot were transplanted with a topsoil and vermiculite ratio of 3:1, and were kept under a controlled environment of 22 °C ± 2 °C temperature and under ~57 µmole photons m^−2^ s^−1^ and 16/8 h light/dark cycles until they were 28 days old. Next, the 28-day-old plants were divided into two groups: Control and 200 mM NaCl Stress. Amounts of 400 mL of 200 mM NaCl and 400 mL of water were applied, and the plans were transferred to different temperature regimes (4 °C, 22 °C, and 31 °C) during the daytime. Leaf samples for RNA Extraction were collected in liquid nitrogen after 8 h of temperature and salt stress application in triplicate. For RNA extraction, TRIzol reagent was used, and 100 mg leaf samples were ground and re-suspended in 1 mL TRIzol. The samples were vortexed for 10 min at room temperature and then centrifuged at 12,000 rpm for 2 min, and the supernatant was transferred to fresh tubes containing 200 µL chloroform. The tubes were vortexed again for 10 s and left on ice for 5 min. The samples were centrifuged again at 12,000 rpm for 15 min, and the supernatant was transferred to fresh tubes. An amount of 300 µL of isopropanol was added to the tubes containing supernatant. The tubes were then mixed gently by inversion and centrifuged for 10 min; after centrifuge the supernatant was discarded without disturbing the RNA pellet. A solution of 70% ethanol was used for washing the RNA pellet. The RNA was re-suspended in 50 µL nuclease-free water, and the samples were incubated in a Dry Oven at 66 °C for 30 min. The quantity and quality were determined using a NanoDrop (NanoQ Optizen, Daegu, Republic of Korea). Moreover, cDNA was synthesized using 2 µg of RNA using a DiaStarTM reverse transcriptase kit (SolGent Co., Ltd, Daejeon, Republic of Korea).

### 4.9. qRT-PCR Analysis

qRT-PCR analysis of *FAD3*, *FAD7*, and *FAD8* was conducted on Bio-Rad CFX Duet Real-Time PCR System by using the synthesized cDNA with a DiaStarTM reverse transcriptase kit (SolGent Co., Ltd, Daejeon, Republic of Korea). For each gene, three replicates were taken, and each replicate contained 20 µL reaction with 10 µL SolgTM 2X Real-Time PCR Smart Mix (Including SYBR Green, SolGent Co., Ltd.), 1 µL Forward Primer, 1 µL Reverse Primer, 7 µL ddH_2_O, and 1 µL of cDNA. ACTIN 12 (Supplementary Table S1) was used as a reference gene with an annealing temperature of 59 °C. Relative gene expression was normalized using the 2^−ΔΔCt^ method with significance at *p* < 0.05. GraphPad Prism 10.5.0.774 software was used for statistical analysis of qRT-PCR expression data. Data were analyzed using two-way ANOVA followed by Tukey’s multiple comparison test at *p* < 0.05.

### 4.10. Identification of Conserved Promoter Motifs and Their Comparative Analysis

Conserved promoter motifs were identified by using a 1000 bp upstream promoter sequence of *AtFAD3*, *AtFAD7* and *AtFAD8* by using MEME (Multiple EM for Motif Elicitation). Comparative analysis was carried out using GraphPad Prism 10.5.0.774 by creating a scatter plot by taking the Y-axis for frequency of identified promoter motifs and the X-axis for gene expression data of *AtFAD3*, *AtFAD7* and *AtFAD8* under control and salt stress conditions at different selected temperatures.

### 4.11. Pearson Correlation Analysis

Pearson correlation analysis was conducted between the identified motifs in the promoter regions and the q-RT PCR data of *AtFAD3*, *AtFAD7*, and *AtFAD8*. Pearson correlation analysis was conducted on GraphPad Prism 10.5.0.774 by computing r for every pair of Y data sets (correlation matrix) and with 95% confidence level.

## 5. Conclusions

In this study, we carried out a comprehensive in silico analysis of the *FAD3*, *FAD7*, and *FAD8* genes across seven different plant species, followed by q-RT-PCR analysis of omega-3 fatty acid desaturase genes (*FAD3*, *FAD7*, and *FAD8*) in *Arabidopsis thaliana* Col-0 under combined temperature and salt stress. Our results showed that all three omega-3 fatty acid desaturase genes are highly conserved in their protein sequences, motifs, and predicted 3D structures. In particular, the histidine-rich domains play an important role in unsaturated fatty acid biosynthesis.

Phylogenetic analysis revealed that the *FAD3* gene is clearly distinct from *FAD7* and *FAD8* in subcellular localization, and the *FAD7* and *FAD8* genes are clustered closely together, confirming their common evolutionary origin and cellular localization. Gene structure also revealed a high degree of similarity in the number of exons and introns. Cis-regulatory elements revealed the abundance of light-responsive elements and ABA-responsive cis elements, suggesting that these genes are strongly regulated by environmental signals. From q-RT PCR analysis, we can conclude that under combined temperature and salt stress, the transcriptional levels of omega-3 fatty acid desaturase (*FAD3*, *FAD7*, and *FAD8*) are strongly influenced in *Arabidopsis thaliana*. The *FAD8* gene exhibited higher expression levels under salt stress conditions, especially at 22 °C, indicating a possible central role in stress adaptation. Pearson correlation analysis and comparative analysis provide statistical evidence for the dynamic shift in gene regulation across different environments, such as that ABA-mediated pathways dominate adaptation at low temperatures (4 °C), whereas drought and salt-responsive motifs drive responses at higher temperatures (31 °C). Overall, these findings suggest that omega-3 fatty acid desaturases are highly conserved yet transcriptionally dynamic. However, these specific promoter-driven regulatory mechanisms offer a promising strategy for researchers aimed at enhancing crop tolerance to salinity and temperature stress.

## 6. Future Recommendations

Based on the conducted analysis, we recommend that researchers check the responses of omega-3 fatty acid desaturase (FAD3, FAD7, and FAD8) knockout mutants and overexpression mutants in comparison with wild type under combined salt and temperature conditions. Furthermore, exogenous application of ABA in MS media and in soil should be performed on knockout mutants and overexpression mutants to understand the responses of mutant lines to ABA application. Moreover, to further support the regulation of omega-3 fatty acid desaturase gene expression under combined salt and temperature stress conditions, a multiple time-point gene expression analysis should be conducted using RNA extracted from both shoots and roots separately to check the expression levels of FAD3, FAD7, and FAD8 in both shoots and roots under combined salt and temperature stress conditions, and taking multiple reference genes for comparison. In addition, KCl should be used as a control in future studies to help differentiate ionic effects from osmotic stress.

## Figures and Tables

**Table 1:** Physicochemical attributes of ER-localized and chloroplast-localized omega-3 fatty acid desaturases from seven different plant species.

Plant Species	Accession Number	Species	M. Weight (da)	Instability Index	Aliphatic Index	Hydropathicity (GRAVY)	Length	pI
AtFAD3	NP_180559.1	*Arabidopsis thaliana*	44,076.72	26.76	92.41	−0.088	386	8.47
AtFAD7	NP_187727.1		51,174.49	34.66	85.7	−0.30	446	8.15
AtFAD8	NP_196177.1		50,136.44	38.91	76.67	−0.318	435	8.74
OsFAD3-1	LOC_Os11g01340.4	*Oryza sativa*	47,540.56	40.25	86.89	−0.080	412	7.82
OsFAD7	LOC_Os03g18070.1		51,373.17	53.55	83.94	−0.215	459	9.66
OsFAD8	LOC_Os07g49310.1		47,011.98	44.75	85.52	−0.160	413	8.81
GmFAD3	Glyma.14G194300.1	*Glycine max*	43,944.61	35.87	90.72	−0.142	376	8.74
GmFAD7	Glyma.18G202600.1		51,247.81	32.78	88.01	−0.168	453	8.45
GmFAD8-1	Glyma.01G120400.1		51,376.92	38.56	87.94	−0.177	452	7.40
ZmFAD3	Zm00001d044605	*Zea mays*	46,145.66	38.79	82.69	−0.291	402	8.23
ZmFAD7	Zm00001d047743		49,385.72	46.37	85.51	−0.176	443	9.16
ZmFAD8	Zm00001d007228		45,977.96	43.75	91.50	−0.036	408	9.00
SlFAD3	Solyc06g007130.2	*Solanum lycopersicum*	43,959.96	30.98	90.69	−0.142	377	8.9
SlFAD7	Solyc06g051400.2		49,659.73	38.93	83.59	−0.223	435	7.78
BnFAD3	WOP78674.1	*Brassica napus*	43,906.37	30.85	91.10	−0.101	383	7.44
BnFAD7	ACS26170.1		49,520.9	29.1	86.64	−0.203	432	7.8
BnFAD8	ACS26171.1		49,463.89	32.38	83.26	−0.225	432	8.57
VuFAD3	ABY60736.1	*Vigna unguiculata*	43,234.05	34.52	87.76	−0.119	371	7.18
VuFAD7	ABY60738.1		51,387.79	32.81	86.28	−0.23	454	8.22
VuFAD8	ABY60737.1		51,395.78	39.84	84.04	−0.232	451	7.13

## Data Availability

The original contributions presented in this study are included in the article. Further inquiries can be directed to the corresponding authors.

## Supplementary Materials

The following supporting information can be downloaded at: https://www.mdpi.com/article/10.3390/ijms27093877/s1.

## Abbreviations

The following abbreviations are used in this manuscript:

ALA	Alpha-linolenic acid
TFAs	Trienoic fatty acid
ER	Endoplasmic reticulum
PUFA	Polyunsaturated fatty acid
FAD	Fatty acid desaturase
