# Supplementary material for: Molecular Characterization of Omega-3 Fatty Acid Desaturases Reveals Functional Conservation and Their Pivotal Role in Salt and Temperature Stress Adaptation in Arabidopsis thaliana
Source: Int J Mol Sci. 2026 Apr 27;27(9):3877. doi: 10.3390/ijms27093877 (PMC13164332; doi:10.3390/ijms27093877)
Supplement: Supplementary file 1 [file ijms-27-03877-s001.zip › ijms-4224950-supplementary.pdf]

Supplementary Table S1. Primers of *Arabidopsis thaliana* omega-3 fatty acid desaturase genes used for quantitative real-time PCR analysis

**Oligo Name**		**Sequence (5' - 3')**
AtFAD8	F	AATTGGCTTCTCTGGCCTCT
	R	GATGACCAGCCACACTGTTC
AtFAD7	F	CGTCGCTATCGTCTTTGCAT
	R	AGATGACCGACCACACTGTT
AtFAD3	F	CTGGGCCATCTTTGTTCTCG
	R	GTGTGTCCGGTGGCTTATTC
AtActin12	F	AGACCAGCTCTTCCATCGAG
	R	GCAGCTTCCATTCCCACAAA
